# Calcium Dynamics in Astrocytes During Cell Injury

**DOI:** 10.3389/fbioe.2020.00912

**Published:** 2020-08-27

**Authors:** Nicole M. Wakida, Veronica Gomez-Godinez, Huayan Li, Jessica Nguyen, Edward K. Kim, Joseph L. Dynes, Shivashankar Othy, Alice L. Lau, Peng Ding, Linda Shi, Christopher Carmona, Leslie M. Thompson, Michael D. Cahalan, Michael W. Berns

**Affiliations:** ^1^Beckman Laser Institute & Medical Clinic, University of California, Irvine, Irvine, CA, United States; ^2^Institute of Engineering in Medicine, University of California, San Diego, San Diego, CA, United States; ^3^Department of Physiology and Biophysics, School of Medicine, University of California, Irvine, Irvine, CA, United States; ^4^Department of Psychiatry and Human Behavior, University of California, Irvine, Irvine, CA, United States; ^5^Institute for Memory Impairments and Neurological Disorders, University of California, Irvine, Irvine, CA, United States; ^6^Department of Neurobiology and Behavior, University of California, Irvine, Irvine, CA, United States; ^7^Sue and Bill Gross Stem Cell Research Center, University of California, Irvine, Irvine, CA, United States; ^8^Institute for Immunology, University of California, Irvine, Irvine, CA, United States; ^9^Department of Biomedical Engineering, University of California, Irvine, Irvine, CA, United States; ^10^Department of Developmental and Cell Biology, School of Biological Sciences, University of California, Irvine, Irvine, CA, United States

**Keywords:** astrocyte, calcium, Salsa6f, phagocytosis, photolysis, laser nanosurgery, laser ablation

## Abstract

The changes in intracellular calcium concentration ([Ca^2+^]) following laser-induced cell injury in nearby cells were studied in primary mouse astrocytes selectively expressing the Ca^2+^ sensitive GFAP-Cre Salsa6f fluorescent tandem protein, in an Ast1 astrocyte cell line, and in primary mouse astrocytes loaded with Fluo4. Astrocytes in these three systems exhibit distinct changes in [Ca^2+^] following induced death of nearby cells. Changes in [Ca^2+^] appear to result from release of Ca^2+^ from intracellular organelles, as opposed to influx from the external medium. Salsa6f expressing astrocytes displayed dynamic Ca^2+^ changes throughout the phagocytic response, including lamellae protrusion, cytosolic signaling during vesicle formation, vesicle maturation, and vesicle tract formation. Our results demonstrate local changes in [Ca^2+^] are involved in the process of phagocytosis in astrocytes responding to cell corpses and/or debris.

## Introduction

There is growing appreciation of the functional capabilities of astrocytes, as recent studies demonstrate their participation in previously overlooked cellular processes like synapse remodeling, axonal guidance (Sofroniew and Vinters, 2010), axonal regeneration (Anderson et al., 2016), blood brain barrier control (Morrison et al., 2011), and phagocytosis (Wakida et al., 2018). It is becoming increasingly evident that astrocytes, which represent 60% of the cells in the human central nervous system (CNS), function as the gate-keepers of the CNS immune responses (Liddelow and Hoyer, 2016). The ability of astrocytes to engulf the debris of apoptotic cells has been demonstrated previously in mixed cortical cultures (Sokolowski et al., 2011), and in human induced pluripotent stem cells (hiPSC’s) differentiated into astrocytes (Tcw et al., 2017).

Recent evidence indicates that astrocyte-mediated phagocytosis is involved in various functions related to neurologic maladies such as Parkinson’s disease (Morales et al., 2017), stroke (Pengyue et al., 2017), Alzheimer’s disease (Gomez-Arboledas et al., 2018), Amyotrophic Lateral Sclerosis (Tripathi et al., 2017), traumatic brain injury (TBI) (Jiang et al., 2018), pain (Chen et al., 2018), and sleep (Bellesi et al., 2017). Developmentally, in *Drosophila* larvae, astrocytes appear to specifically transform into phagocytes that are the primary cell types involved in the pruning and clearance of synaptic and neural debris during metamorphosis (Tasdemir-Yilmaz and Freeman, 2014). In mammals, for example, it also has been shown that astrocytes have a major protein synaptic pruning function via active engulfment (phagocytosis) of over abundant CNS synapses (Chung et al., 2013).

Despite the studies clearly implicating astrocytes as modulators of neural repair and synaptic pruning via phagocytosis (Chung et al., 2013), little is known about the cellular-based ionic signaling connected with these processes. Whereas recent research is revealing direct impact of astrocyte-related intracellular changes in calcium [Ca^2+^] in diverse neurologic areas such as neural circuit plasticity and synchronization, downstream effects on cellular circuits are not well understood (Guerra-Gomes et al., 2017). Recent studies on microglia focusing on the ER Ca^2+^ sensor STIM1 and the plasma membrane Ca^2+^ channel Orai1, suggest that chronic and global Ca^2+^ regulate such functions as release of cytokines and gliotransmitters including ATP, and phagocytosis (Lim et al., 2017; Toth et al., 2019). Since activated astrocytes are now known to initiate phagocytosis to remove damaged and dead cells in a similar fashion to microglia, it is likely that astrocytes also exhibit Ca^2+^ modulated regulation of phagocytosis (Loov et al., 2012; Wakida et al., 2018).

In our previous study we described the utilization of laser nanosurgery/ablation to induce catastrophic damage resulting in rapid cell death of a single astrocyte or neuron (Wakida et al., 2018). Nearby non-irradiated astrocytes became activated to phagocytose the dead cell debris. In that study we characterized the cytological and behavioral changes of the responding astrocyte as it interacted with the dead cell or its debris. The phagocytic process involved extensive endocytic vesicle formation during the process of phagocytosis by the activated astrocyte. The value of our overall approach is the ability to study astrocyte responses, including phagocytosis, at the single cell level. In the study reported here, we describe changes in intracellular [Ca^2+^] in adjacent astrocytes responding to cell damage and death.

We employ subcellular laser ablation to lyse individual astrocytes (photolysis) while monitoring changes in [Ca^2+^] in adjacent non-damaged astrocytes. These studies are conducted in several different astrocyte systems: (1) the Ca^2+^ sensitive dye Fluo4 loaded into primary astrocytes isolated from mouse brain tissue, (2) the Ca^2+^ sensitive dye Fluo4 in the established astrocyte cell line Ast1, and (3) astrocytes derived from brain cortex of mice endogenously expressing the ratiometric genetically encoded Ca^2+^ indicator (GECI), Salsa6f (Dong et al., 2017). Imaging of astrocytes during this process demonstrate a link between Ca^2+^ signaling and astrocyte response to neural cell damage. Salsa6f labeled astrocytes provide clear evidence of dynamic changes in Ca^2+^ at the subcellular level, where local increases in Ca^2+^ correspond to progressive steps of phagocytosis. These results demonstrate a direct relationship between Ca^2+^ and astrocyte response to injury.

## Materials and Methods

### Ca^2+^ Fluorophores and Tissue Sources

Salsa6f astrocytes were derived from brain cortex of mice expressing GFAP-Cre Salsa6f. Salsa6f is a genetically encoded ratiometric Ca^2+^ indicator derived from the fusion of GCaMP6f and td-Tomato, as described in Dong et al. (2017). Adult mouse primary astrocytes were cultured according to the protocol outlined by Sun et al. (2017). Cells were dissociated from the cortex, striatum, and hippocampus. They were plated on gelatin-coated glass bottom 35 mm imaging dishes (Mattek Corp) and carried in DMEM with 20% FBS, Forskolin, and GDNF additives. Cells were incubated at 37°C at 5% CO_2_. Cells were imaged in DMEM with no phenol red, 10% FBS, N_2_, Forskolin, GDNF, and gentamycin/amphotericin B.

GFAP positive primary astrocytes (via antibody staining) from embryonic (E18) mouse cortex (Brain Bits) cultures were established as outlined in our previous publication (Wakida et al., 2018). Cultures of immortalized GFAP-positive astrocyte type 1 cell line (Ast1) acquired from ATCC were also used, as outlined previously (Wakida et al., 2018). Both primary astrocytes and immortalized astrocyte cells were labeled with Fluo4 AM (Thermo Fisher) Ca^2+^ indicator. Cells were washed with HBSS once, prior to the addition of 100 nM Fluo4 and 2.5 mM Probenecid (inhibitor of organic-anion transporters in cell membrane) diluted into HBSS or Silac imaging media. Cells were incubated with Fluo4/Probenecid for 30 min prior to imaging.

This study was carried out in accordance with the principles of the Basel Declaration and recommendations of the University of California, Irvine Institutional Animal Care and Use Committee. The protocol was approved by the University of California, Irvine Institutional Animal Care and Use Committee.

### Ca^2+^ Free Medium

Ca^2+^ free DMEM (Gibco Thermo cat #2106828) was supplemented with sodium pyruvate dissolved in water (Gibco Thermo cat# 11360) and GlutaMAX dissolved in a sodium chloride solution (Gibco Thermo #35050). Serum was omitted from Ca^2+^ free DMEM. A 100 mM stock EGTA solution was made by dissolving EGTA into water with a pH of 11 using NaOH. After dissolution, the 100 mM EGTA solution was pH adjusted to 7.4 with concentrated HCl. 1 mL of stock EGTA was added per 100 mL of Ca^2+^ free medium.

Ca^2+^ free experiments were first performed on glass bottom imaging dishes (Cell E&G # GBD00004-200). Cells were grown in regular Ca^2+^ containing medium as described above. Prior to experiments cells were washed with HBSS without Ca^2+^ or Mg^2+^ and then bathed in Ca^2+^ free medium. In later experiments medium replacements were done through perfusion for immediate visualization. This was achieved by seeding Ast1 cells into Rose chambers (Rose et al., 1958). Syringe needles were inserted into opposite sides of a rubber gasket of a 1.5 mL volume Rose chamber. The needles were connected to tubes via needle tubing and a dual male to male luer slip coupler. The opposite side of one of the tubes was coupled to a syringe containing Ca^2+^ free DMEM with 1 mM EGTA. The tubing on the other side was connected to an empty syringe. Cells grown in regular medium were imaged on the microscope prior to perfusion with Ca^2+^ free medium. Perfusion occurred via suction with the empty syringe. This caused the Ca^2+^ medium to be pulled out while the Ca^2+^ free medium flowed into the chamber. Rose chambers hold approximately 1.5 mL of solution. For effective medium replacement 10 mL of medium was pulled out of the syringe carrying the Ca^2+^ free EGTA-MEM. Perfusion of Salsa6f cells was done utilizing a 35 mm dish insert from Warner Instruments (RC-37). Tubing was connected to syringes where one was used to pull out the solution while the other syringe was pressed to release Ca^2+^ free medium.

### Optical Systems and Parameters

Two Zeiss Axiovert 200M inverted microscopes with 40 X, NA 1.3 objectives combined with a Hamamatsu Orca-R_2_ cooled CCD cameras were used to acquire phase contrast and fluorescence images. Cells were incubated at a controlled environment of 37°C, 5% CO_2_, and 70% humidity using a temperature and gas stage incubation system (Ibidi). Laser induced damage was accomplished using two different short-pulse femtosecond lasers focused by a 40 X, 1.3 NA phase contrast objective to a diffraction-limited spot. One system used a Coherent Mira 900 laser emitting 800 nm pulses of 200 fs pulse width at an emission rate of 76 MHz. The second system was a Mai Tai Spectra Physics laser also emitting 76 MHz pulses at a wavelength of 780 nm. In both systems laser power was attenuated with a Glan-Thompson polarizer to a minimal irradiance sufficient to result in rapid death of the targeted cell. The amount of laser pulses in the target spot was regulated by an Oriel shutter and Uniblitz shutter controller which had an “open” exposure duration time of 10–40 ms. The position of the laser was directed by a fast scanning mirror as described previously (Duquette et al., 2018). All components in both systems were controlled through custom Robolase software, which allows for automation and integration of laser and imaging components (Botvinick and Berns, 2005).

Salsa6f fluorescence imaging was accomplished using the built-in filter turret in the Zeiss Axiovert 200M with filter sets for GFP and Cy3 (Chroma). Phase contrast images were acquired at 10 s intervals. Subsequent green and red fluorescence images were taken at approximate 2 min intervals (average 1 h 45 min observation time). In some experiments the green GCamp6f signal from Salsa6f emission was plotted and in others, a green: red ratio of both the GCaMP6f and td-Tomato wavelengths was plotted (see *Y* axis labeling of graphs).

Shorter time-frame imaging of Salsa6f fluorescence was accomplished using an excitation filter and dichroic mirror set ZET 488/561x (Chroma) that allows for simultaneous excitation of GFP and tdTomato fluorophores using an EXFO X-Cite 120 fluorescence illuminator. A motorized emission filter wheel driven by Mac 6000 interface module (LEP) capable of 50 ms change was used to switch between green emission filter HQ 525-50 and red emission filter HQ 572 LP (Chroma). Green and red fluorescent images were acquired at 2 s intervals. The image acquired immediately following photolysis was approximately 5 s following laser shutter closure. For control cells, the laser was fired in a region vacant of cells, but adjacent to cultured astrocytes. Laser parameters were consistent with those used to kill cells by laser exposure. Ca^2+^ signal was measured in the cells surrounding the laser-targeted control region.

### Image Analysis

To quantify relative Ca^2+^ changes, Image J was used to define a region of interest (ROI) within individual cells. Average fluorescence pixel intensity was determined for each ROI throughout the image sequence. Traces corresponding to relative Ca^2+^ change (background subtracted) were graphed with Microsoft Excel.

To calculate the changes in fluorescence intensity before vs. after laser exposure, the average pixel intensities of an ROI specific to each cell was used to determine average pixel intensity for each image using Image J. Change in fluorescence value DeltaF/F (dF/F) was calculated by subtracting pre fluorescence intensity (before photolysis) from post fluorescence (image acquired immediately following photolysis), and dividing by pre fluorescence intensity. Green red ratio (G/R ratio) images processed with the image calculator function of ImageJ, with LUT fire for image pseudo color, where the brightest pixel intensities correspond to orange shades, and dimmest pixel intensities correspond to blue shades. For Salsa6f cells, dF/F was calculated from the green GCaMP6f signal. Ratio images were not used due to the large number of images taken over this short period of time, resulting in substantial photobleaching in the red channel of cells that were non-motile during the observed period.

For T_1__/__2_ calculations of Salsa6f and Fluo4 signals, the time it took for the cell to drop from maximum pixel intensity to half the value of maximum pixel intensity minus the minimum pixel intensity at the start of the increase in [Ca^2+^] (initiation of the peak) was determined.

## Results

### Ca^2+^ Dynamics in Astrocytes

Here we describe an *in vitro* cellular model of astrocyte phagocytosis that allows for the acquisition of fluorescence and corresponding phase contrast images over an extended imaging period. Utilization of laser ablation to mimic a brain injury event (photolysis) permits the precise control of timing and location of cell death, and thus allows direct comparison of cells before and after cell injury. Additionally, the amount of damage induced was limited to a single cell, allowing for the observation of varying events occurring within the visible network of cells.

**FIGURE 1:**
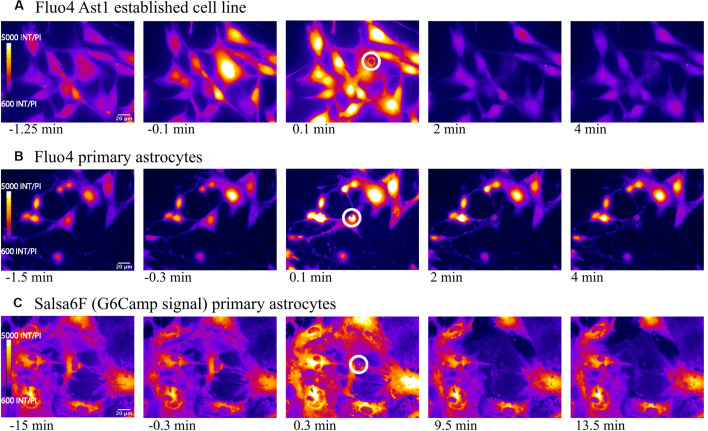
Elevated cytosolic Ca^2+^ signal in response to photolysis. Ca^2+^ elevation is observed throughout astrocyte networks following photolysis of a central cell, using 2 different Ca^2+^ indicators, Fluo4 **(A)** and Salsa6f **(B,C)**. Astrocytes were derived from 2 sources, an established astrocyte line in **(A)** and mouse primary cortical astrocytes in **(B,C)**. The blue arrow in the third column delineates the laser irradiated region depicted by the white ROI. By 2 min post irradiation, fluorescence in uninjured astrocytes returns to baseline levels observed prior to laser exposure.

We first compared the widely used Ca^2+^ indicator Fluo4, with the genetically encoded Ca^2+^ indicator GECI Salsa6f, during visualization of intracellular Ca^2+^ changes in an astrocyte population exposed to laser ablation. Figure 1 displays a representative field of the 3 types of Ca^2+^ labeled astrocytes: (1) Fluo4 in the established Ast1 astrocyte cell line, (2) Fluo4 in primary astrocytes isolated from mouse cortex, and (3) astrocytes derived from the cortex of mice expressing the Ca^2+^ sensitive fluorescent GFAP-Cre Salsa6f probe. Images in Figure 1 display changes in fluorescence intensity for single wavelength indicator Fluo4 vs. GCaMP6f channel of Salsa6f. All images are pseudo colored with LUT fire, where the brightest pixel intensities correspond to orange shades, and dimmest pixel intensities are in blue. Baseline imaging of the cells prior to the laser-induced targeted cell death (photolysis), demonstrates moderate Ca^2+^ changes within resting astrocytes, within frames 1–2, 6–7, and 11–12 in columns 1 and 2. Astrocytes demonstrate spontaneous Ca^2+^ flashing and waves that propagated through the astrocyte network, visible in time lapse movies prior to laser perturbations in both Salsa6f and Fluo4 labeled astrocytes (Supplementary Videos S1–S3). The laser is exposed to the target at time 0; the target position is depicted by the highlighted white ROI in Figure 1 frame 3, 8, and 13.

In all three cell sources, an elevation in Ca^2+^ signal was apparent throughout the astrocyte network immediately following laser ablation of a central cell (Figure 1, blue arrow demarcates position of laser irradiation displayed in column 3). This elevation diminishes over time returning to baseline levels in all astrocyte models, evident in fourth and fifth columns of Figure 1 and Supplementary Videos S1–S3 (Supplementary Video S1: Fluo4/Ast1, Supplementary Video S2: Fluo4/primary astrocytes, Supplementary Video S3 Video Salsa6f/primary astrocytes).

### Cytoplasmic Ca^2+^ Elevation in Response to Laser Damage

**FIGURE 2:**
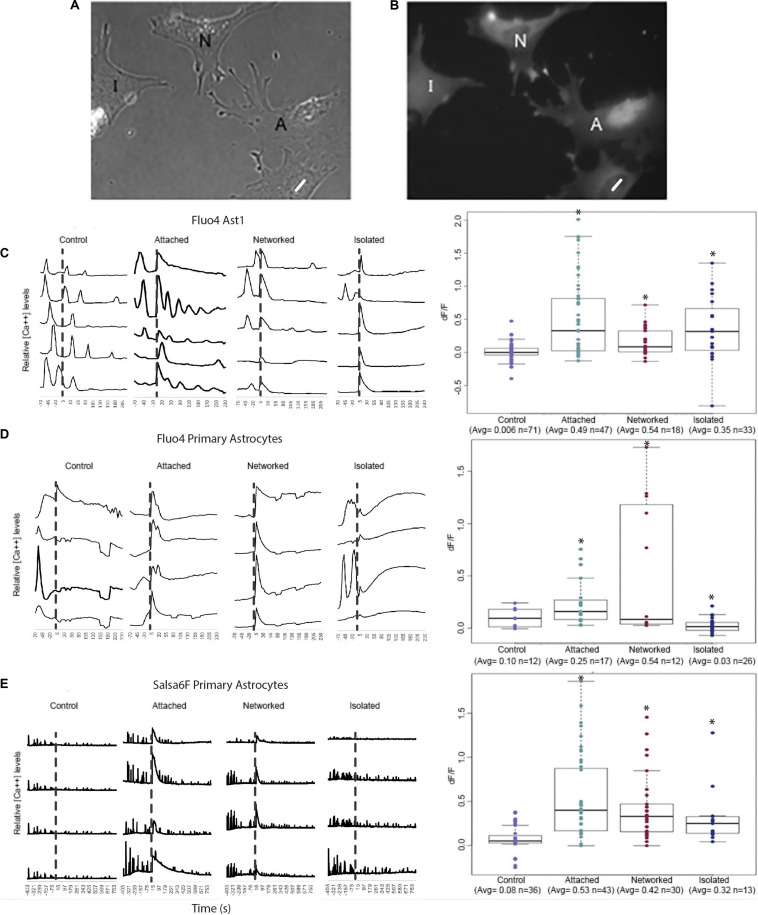
Quantification of Ca^2+^ elevation within the astrocyte network. Astrocytes were categorized into 4 groups: control, attached, networked, and isolated. **(A,B)** Phase contrast **(A)** and corresponding Ca^2+^ fluorescence signal **(B)** of a representative group of astrocytes depicting attached (A), networked (N), and isolated (I) astrocytes with respect to photolyzed cell (white ROI). Attached astrocytes shared a membrane with the photolyzed cells, networked cells were indirectly connected to the photolyzed cell, and isolated cells shared no membranous connection with the photolyzed cell. **(B–D)** Ca^2+^ signal traces of control, attached, networked, and isolated astrocytes and dF/F in response to photolysis of a neighboring cell. Representative traces are shown for Fluo4 labeled Ast1 cells **(C)**, Fluo4 labeled primary astrocytes **(D)**, and Salsa6f labeled primary astrocytes **(E)**. Control astrocytes did not respond to the laser being fired within an adjacent area vacant of cells, but did show spontaneous Ca^2+^ spiking throughout the observation period. A significant Ca^2+^ oscillation is consistently observed throughout the contiguous astrocyte network (attached and networked cells) immediately following the photolysis event, denoted by the dashed vertical line. Isolated astrocytes from the Ast1 cell line also displayed a sharp rise in cytosolic Ca^2+^, with a significant, but less dramatic rise in primary astrocytes labeled with both Fluo4 and Salsa6f. Box plots of dF/F displaying a value for change in Ca^2+^ signal in response to photolysis are displayed for each fluorophore/astrocyte combination. All categories (attached, networked, isolated) showed significant increases in dF/F values when compared to control cells, depicted by asterisk (^∗^) over each column.

For quantitative assessment of Ca^2+^ fluctuations over time, we plotted Ca^2+^ signal intensity for four to five typical cells in 4 categories: (1) control cells where the laser was fired into an area vacant of cells, adjacent to the observed cell (as opposed to on the cell); (2) “attached” cells that share membrane attachments with the laser-killed astrocyte; (3) “networked” cells indirectly connected to the irradiated cell (in contact with cells directly attached to the killed cell); and (4) “isolated” cells neither in contact with the killed cell nor in contact with networked cells (see Figures 2A,B for schematic of 4 categories). Traces for each of the 4 cell categories are shown for 3 types of Ca^2+^ labeled astrocytes: Fluo4 labeled Ast1 (Figure 2C), Fluo4 labeled primary astrocytes (Figure 2D), and Salsa6f labeled primary astrocytes (Figure 2E). Ca^2+^ signal traces are displayed from cells within the same field of view for control, attached, and networked categories. Isolated astrocytes were not prevalent enough within the cultures to image 4 or 5 cells within the same field of view. Thus, isolated traces are derived from cells from two different field of views.

Control astrocytes from all 3 sources (Fluo4 labeled Ast1, Fluo4 labeled primary astrocytes, Salsa6f labeled primary astrocytes) displayed spontaneous transients occurring at random, with no apparent relationship to laser exposure next to cells (time of irradiation depicted by the vertical dashed line in Figures 2C–E). A large, transient oscillation associated with photolysis of an adjacent cell is observed in attached and networked cells for all three sources of cells. This oscillation was also evident in isolated Ast1 astrocytes, however, it was lower in amplitude in primary astrocytes labeled with Fluo4 and Salsa6f. In comparing the Ca^2+^ intensity traces for Fluo4 and Salsa6f, we observed a greater number of peaks in Salsa6f labeled astrocytes vs. Fluo4 labeled astrocytes. The variation in response between Salsa6f and Fluo4 is likely due to the sensitivity of each labeling technique. Fluo4 highlights smaller fluctuations in intracellular Ca^2+^ with larger fluorescence intensity increases. With Fluo4 we observe large increases in Ca^2+^ throughout the entire cell in response to the same induced damage. GCaMP6f is an ultrafast Calmodulin-based indicator beneficial at detecting low threshold, fast rising oscillations. GCaMP6f can monitor oscillations up to 20 Hz (Li et al., 2019). with a T_1__/__2_ rise as fast as 2 ms and T_1__/__2_ decay time of 63 ms at 37°C (Helassa et al., 2016). The Salsa6f probe shows changes within intracellular regions of a cell, with smaller Ca^2+^ intensity increases (Refer to Supplementary Video S3).

The transient fluorescence response following laser irradiation compared to baseline intensity (prior to laser irradiation) was calculated as a *dF/F value. dF/F* and number of sample observations (n) for the different cell categories and sources are displayed in box plots to the right of Ca^2+^ traces in Figure 2. All groups demonstrated statistically significant different dF/F values between control cells and all categories of cells (attached, networked, and isolated; see Supplementary Table S1) and depicted by the asterisks in Figures 2C–E. For Fluo4 labeled Ast1 astrocytes, no significant difference was observed when isolated cells were compared to attached and networked categories. This was the opposite of the trend observed in primary cultured cells, where the average dF/F value for attached and networked categories were significantly higher than observed in isolated astrocyte response (attached = 0.53, networked = 0.42, isolated = 0.32, control = 0.08). The decrease in dF/F of Fluo4 labeled isolated cells in response to the photolysis event is likely due to the lack of gap junctions that normally link an astrocytic network together physically. The smaller dF/F value could also be due to a delayed response of Fluo4 isolated astrocytes. A trend of slowly increasing Ca^2+^ signal can be seen in response to photolysis, with Ca^2+^ fluorescence continuing to increase over the observation time. This is especially visible in traces from primary isolated astrocytes labeled with Fluo4. Isolated astrocytes may need additional time for signaling to reach a threshold for a significant increase in fluorescence to be observed.

In both Fluo4 and Salsa6f cells, Ca^2+^ transients were detected in cells surrounding the photolyzed cell. There were some variations between the different Ca^2+^ indicators, mainly that Fluo4 labeled cells did not show a significant difference between the attached and networked primary astrocyte populations whereas Salsa6f labeled cells did (Refer to Supplementary Table S1). When comparing Fluo4 and Salsa6f labeling of primary cells, Salsa6f demonstrated a significantly lower dF/F average value for networked cells in comparison to attached cells (*p* > 0.01). For Fluo4 cells, no detectable difference in dF/F was detected between attached (average = 0.53) and networked primary astrocytes (average = 0.42). The dF/F in Salsa6f cells decrease with the relative location from the photolyzed cell increased (attached = 0.53 > networked = 0.42 > isolated = 0.32).

### Exponential Decay of Ca^2+^ Oscillation in Salsa6f Labeled Astrocytes

**FIGURE 3:**
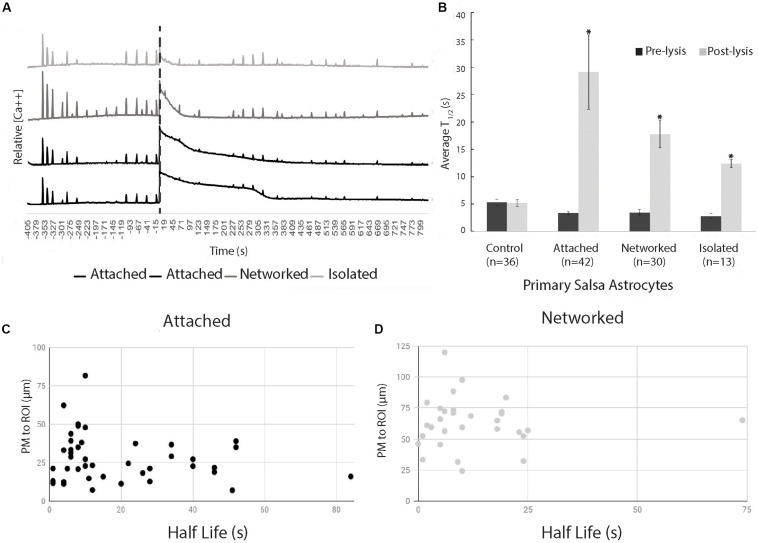
Exponential decay of Ca^2+^ oscillation following photolysis event. **(A)** Representative Ca^2+^ signal traces for 2 attached, 1 networked, and 1 isolated astrocyte labeled with Salsa6f. The large Ca^2+^ oscillation coinciding with the time of photolysis (depicted by vertical dashed line), diminishes at varying rates dependent on the cell’s relative location to the photolyzed cell. **(B)** Comparison of average time to 50% decrease from the peak of signal, *T*_1/2_, decay of transients before photolysis (Pre-lysis) and Ca^2+^ oscillation at time 0/photolysis (Post-lysis). Average pre-lysis *T*_1/2_ for all cell categories ranged between 2.8 and 5.3 s. Attached astrocytes displayed the longest average for post-lysis *T*_1/2_, at 29 s. Networked and isolated categories displayed lower *T*_1/2_ average values at 18 and 12 s, respectively. Post-lysis *T*_1/2_ for all categories except control had a significantly longer post-lysis *T*_1/2_ when compared to pre-lysis *T*_1/2_ values, depicted by asterisks. **(C,D)** Scatter plot of *T*_1/2_ vs. distance of responding cell to laser-targeted region for attached **(C)** and networked **(D)** astrocytes. We observe no correlation between *T*_1/2_ and distance between laser damage (ROI) and closest plasma membrane (PM) of the observed/responding cell for both attached (*r*^2^ = 0.033) and networked cells (*r*^2^ = 0.00039).

The initial Ca^2+^ oscillation in response to neighboring cell death appears to decay exponentially (*R*^2^ = 0.90), but varied in length of time of the decay period (Figure 3). Figure 3 displays a comparison of time for a 50% decrease (*T*_1__/__2_) calculated for two time periods: pre laser control exposure and post laser exposure for each cell category: control, attached, networked, and isolated. Average *T*_1__/__2_ of peaks prior to laser exposure ranged between 2.8 and 5.3 s (control 5.3 s, attached 3.3 s, networked 3.4 s, isolated 2.8 s).

The average time for a 50% decrease (*T*_1__/__2_) from the peak following photolysis (at time 0) of Salsa6f Ca^2+^ oscillation was 29 s (*n* = 42) for the attached cell group, and 18 s (*n* = 30) for networked cells. When spontaneous Ca^2+^ transients occurred in control (*n* = 36) and post-lysis peak in isolated cells (*n* = 13), the *T*_1__/__2_ was 5 and 12 s, respectively (Figure 3). A significant increase was observed between pre and post *T*_1__/__2_ values for all categories (attached *p* < 0.01, networked *p* < 0.01, isolated *p* < 0.01) except for control cells (*p* = 0.93). A significantly higher post-lysis *T*_1__/__2_ was observed for attached, networked and, isolated categories when compared to pre-laser *T*_1__/__2_ values (all *p* > 0.01).

The positions of attached and networked astrocytes relative to the damaged cell vary due to the nature of the defined categories. The average distance between the ROI and attached cells is 28 μm, while the average distance between the ROI and networked cell averaged 63 μm. *In vitro* Salsa6f astrocytes had an average diameter of 46 μm (*n* = 49), similar to the difference between the average distance from ROI to attached and networked cells. To determine if there is a link between the rate Ca^2+^ signal diminishes and the distance from the cell to the laser-targeted region, we have plotted *T*_1__/__2_ values vs. the distance between the laser ROI and the closest point of the plasma membrane of the responding cell. Data points in Figure 3C correspond to attached cells, and data points in 3D correspond to networked cells. We observed no correlation between *T*_1__/__2_ and distance of the responding astrocyte to the targeted laser position, as determined using Pearson’s product-moment correlation coefficient. Attached cells had a *r*^2^ value of 0.033, and networked cells had a *r*^2^ value of 0.00039.

In an interesting observation, a small percentage (less than 5%) of Salsa6f labeled astrocytes responded to photolysis of a neighboring cell with additional and frequent Ca^2+^ spiking. An example of two astrocytes responding to the photolysis of an attached cell can be observed in Supplementary Video S4.

### Origin of Ca^2+^ Transients

**FIGURE 4:**
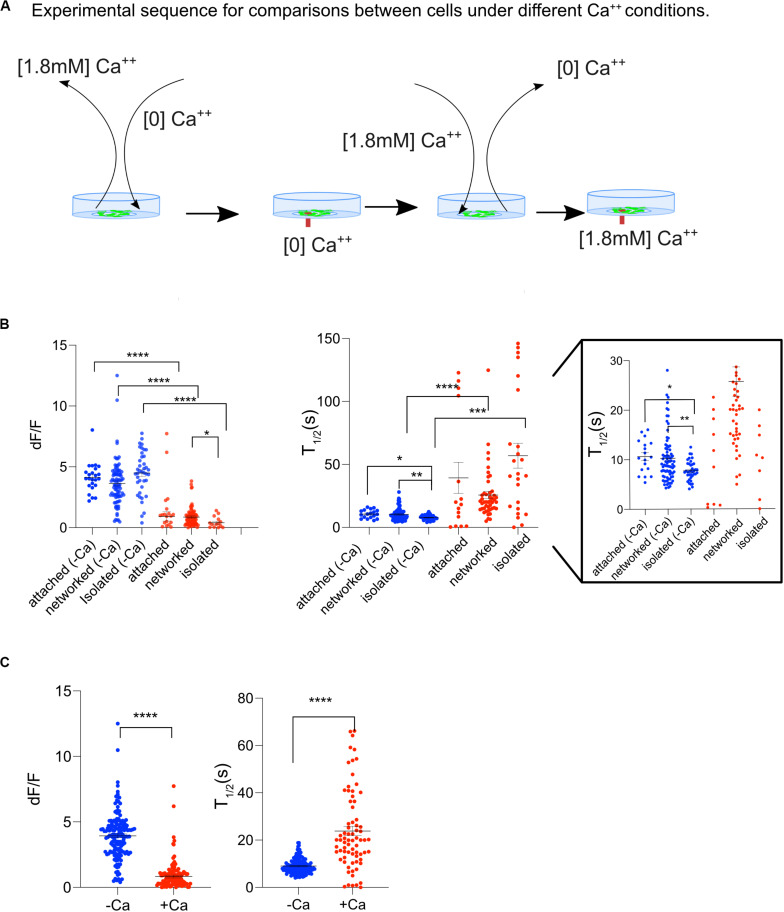
Ast1 Cells in low Ca^2+^ medium display a Ca^2+^ oscillation after single cell photolysis. **(A)** Sequence of events for experiments shown on this figure. Cells grown in regular DMEM were washed with [0] Ca^2+^ HBSS and bathed in low Ca^2+^ DMEM approximately 3 min before placing them on the microscope. A central cell in the field of view was lysed while in low Ca^2+^ DMEM. The field of view was imaged for 5 min before the medium in the dish was replaced with Ca^2+^ containing medium. A second lysis event was triggered within a different field of view of the same dish. **(B)** The dF/F and *T*_1/2_ are shown for Ast1. Each dot is representative of a single cell. Cells were separated into attached, networked and isolated categories. Low calcium was abbreviated as –Ca and normal calcium was abbreviated as +Ca. Blue is used to indicate cells in low calcium medium and red is for cells in regular calcium medium. The *T*_1/2_ is shown with two different *y*-axis ranges. The first range is 0–150 s. The same graph is shown on the right with a range of 0–30 s. Asterisks denote significance. *P ≤ 0.05, **P ± 0.01, ***P ± 0.001, ****P ± 0.0001. **(C)** Combined results from experiments in **(B)**.

To determine whether the transients in cytosolic Ca^2+^ was due to a release of Ca^2+^ from internal cell organelles, or due to an internal influx through the outer cell membrane, Ast1 cells were bathed in commercially available Ca^2+^ free MEM without supplementation of bovine serum (subsequently referred to as low Ca^2+^ medium). The cells were bathed in low Ca^2+^ medium approximately 2 min prior to imaging (Figure 4A). After conducting experiments in low Ca^2+^ medium, the cells were returned to normal Ca^2+^ containing medium to assess the cells responsiveness following a stressful condition of low Ca^2+^. Surprisingly, Ca^2+^ oscillations in low Ca^2+^ medium were 5-fold larger than in regular medium. The average dF/F of cytoplasmic Ca^2+^ transients for cells bathed in low Ca^2+^ was statistically higher (3.9 ± 0.14) compared with the cells in regular DMEM (0.8 ± 0.11), Figure 4B; *n* = 153 and 117 respectively, *p* < 0.0001. These results suggest that intracellular calcium stores are largely responsible for the cytoplasmic increases and that external Ca^2+^ concentrations may affect the degree of Ca^2+^ release. When the data from all cells was combined, cells in low Ca^2+^ medium had a shorter *T*_1__/__2_ than cells in Ca^2+^ medium (9 s vs. 24 s; *n* = 131 and *n* = 74, respectively) (Figure 4C). Isolated cells in low Ca^2+^ had the shortest *T*_1__/__2_ (8 s) when compared to all groups.

**FIGURE 5:**
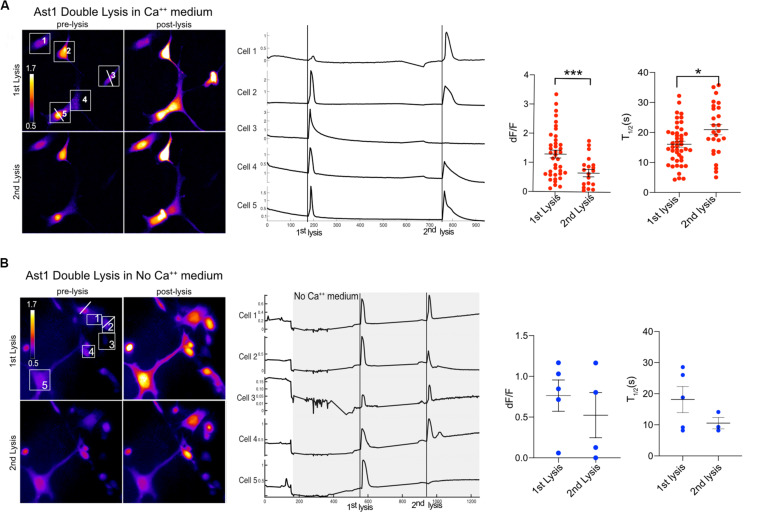
Ast1 cells respond to multiple photolysis events in both Ca^2+^ and no Ca^2+^ conditions. **(A)** Cells in Ca^2+^ free medium (low Ca^2+^ + 1 mM EGTA) were loaded with Ca^2+^ indicator Fluo4. Two cells were sequentially photolyzed within the same field of view. Pseudo color images are shown for the pre and post photolysis. Lysed cells are marked by a white horizontal line ROI through the cell. The numbered ROI have corresponding Ca^2+^ traces to the right. The vertical line in the traces demarcates the time of photolysis within the traces. In this example, cells 3 and 5 were lysed respectively. Experimental data from different fields were combined to generate the dF/F peak and *T*_1/2_ graphs. A *t*-test shows a significant difference in peak response between the 1st and 2nd lysis. ***P < 0.001. *N* = 39 for the 1st lysis and *n* = 20 for the second lysis from combined experiments. A significant difference in *T*_1/2_ between the 1st and 2nd lysis was found, *P ± 0.05. **(B)** Ast1 were perfused with Ca^2+^ free medium prior to photolysis. The first lysed cell is to the left of cell 1 and was not quantified or numbered. The second lysis occurred on cell 2. The Ca^2+^ traces of cells in the field of view are displayed, where the gray area represents the period in which cells are in Ca^2+^ free medium. Perfusion of Ca^2+^ free medium occurred over a period of 200 s. The fluorescence intensity changes during perfusion are do to the changing focus due to the pressure between the coverslips of the rose chambers. The dF/F and *T*_1/2_ values shown correspond to the cells shown in **(B)** and are not combined with other data.

**FIGURE 6:**
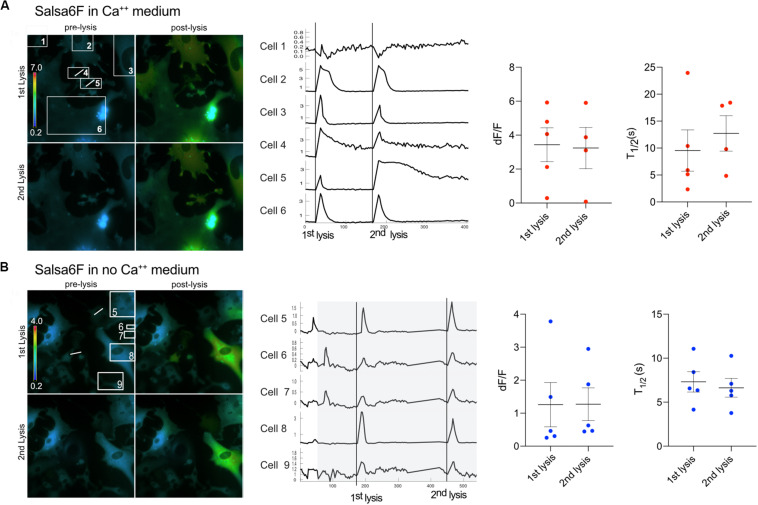
Primary Salsa6f astrocytes respond to multiple lysis events. **(A)** Two cells (4 and 5) were lysed within the field of view shown in the ratiometric images on the left. A white line is drawn across the cell that was photolyzed. Both lysis events lead to cytoplasmic increases within cells in the field of view. Images of the first two lysis events are shown. Where the top row is of the 1st lysis. A pre and post lysis images are shown where the prelysis image contains the regions of interest for each cell. Ca^2+^ traces are shown to the right of the images. dF/F and *T*_1/2_ values are shown for combined data from multiple field of views. **(B)** Cells were perfused with Ca^2+^ free medium for a period of 90 s to minimize cell perturbation. The area in gray depicts the period in which cells are in Ca^2+^ free medium. Cells responded to two lysis events while bathed in Ca^2+^ free medium.

Furthermore, the ability of cells to respond to a second lysis event was tested. We found that Ast1 cells were capable of a second oscillation when in regular Ca^2+^ medium (Figure 5A). Combined results from different experiments shows that the second lysis event induced a smaller oscillation of longer duration than the first, dF/F 0.65 vs. 1.30; *n* = 20 and *n* = 39 respectively. The *T*_1__/__2_ was 16 s for the first lysis event and 21 s for the second event. The ability of cells to respond to a second lysis event was also investigated when cells were perfused with commercial Ca^2+^ free medium supplemented with 1 mM EGTA (see description in section “Materials and Methods”). In these experiments Ca^2+^ free medium is achieved by chelation of any remaining Ca^2+^ in the commercially available low Ca^2+^ medium. Cell cultures were perfused slowly with Ca^2+^ free medium for approximately 3 min. Two single cells within a group of five cells were sequentially killed via photolysis. Cytoplasmic Ca^2+^ transients occurred during both lysis events (Figure 5B). Primary Salsa6f astrocytes also showed the ability to respond to a second photolysis event in both Ca^2+^ and Ca^2+^ free medium (Figures 6A,B, respectively). A time lapse movie corresponding to perfused cells with subsequent photolysis event described in Figure 6B is included as Supplementary Video S5.

### Cytoplasmic Ca^2+^ Elevation in Salsa6f Labeled Astrocytes

**FIGURE 7:**
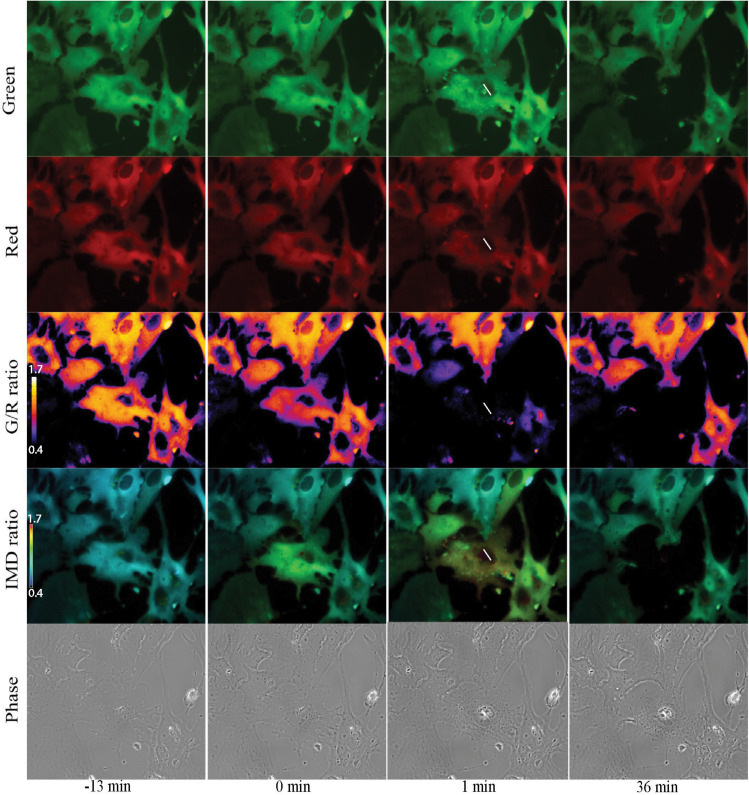
Ratiometric imaging of an astrocyte network in response to photolysis. Green GCaMP6f and red tdTomato images directly acquired during fluorescent imaging are displayed in rows 1 and 2. A small increase in green signal intensity is visible 1 min post photolysis. Fluorescence from the photolyzed cell (white roi) diminishes after photolysis, signal is completely absent in the 36 min post image. Ca^2+^ sensitive green images were divided by Ca^2+^-insensitive red images, and pseudo colored with LUT fire, displayed in row 3. The corresponding scale to LUT fire is overlaid on the pre-lysis/13 min image where large fluorescence intensity changes are indicated by white pixels, decreasing in color warmth to small intensity changes corresponding to dark blue and black pixels. The central lysed cell (white ROI) loses all fluorescence signal within 1 min or photolysis, changing from orange to black hue. Green/red ratiometric images were also analyzed using intensity modulated display (IMD) ratio, displayed in row 4. IMD ratio images highlight changes of pixel intensities observed in cells attached to the targeted cell 1 min following photolysis. Fluorescence levels return toward pre-lysis levels in the 36 min post photolysis image. Phase contrast images in row 5 show minimal changes in the surrounding astrocyte network in response to the photolyzed central cell. The color scale bars for LUT fire and IMD indicate maximum and minimum brightness (BRT) as 5000 and 600, respectively.

**FIGURE 8:**
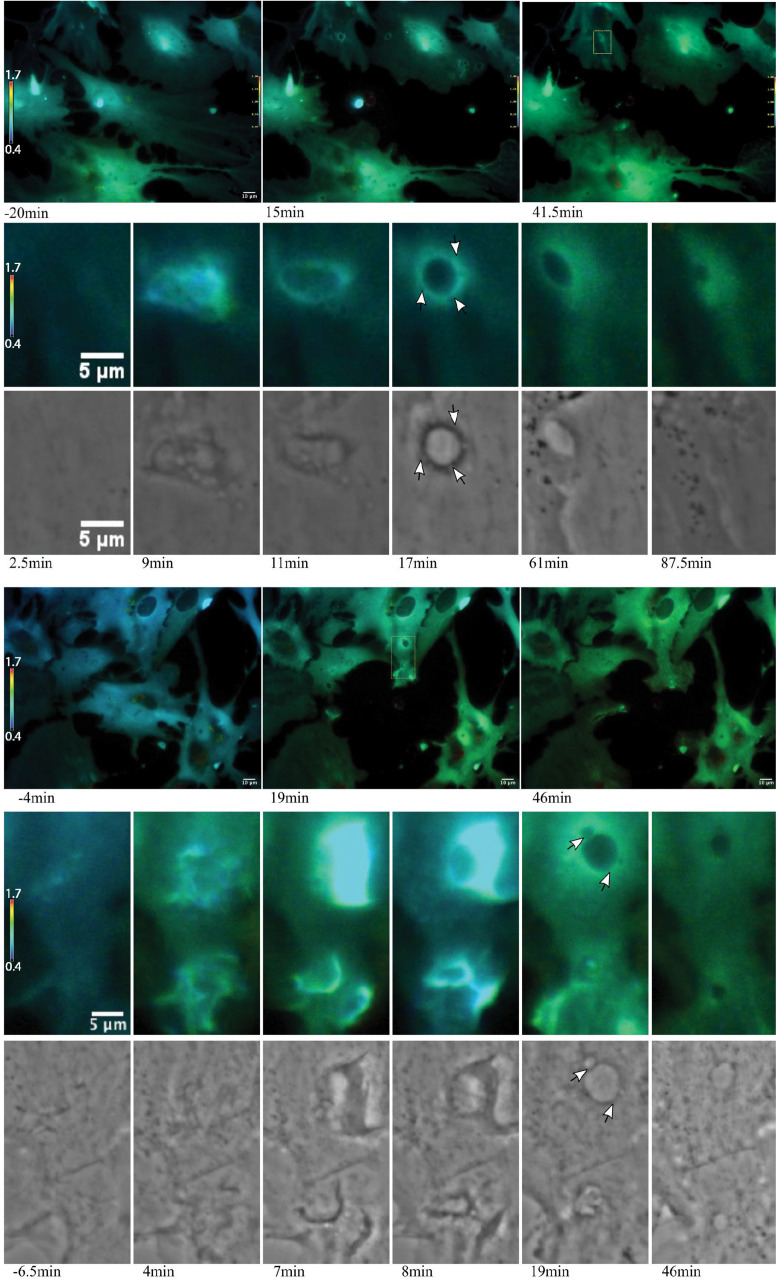
Ca^2+^ localization during endocytic vesicle formation and maturation. Two representative networks of astrocytes respond to photolysis via vesicle formation. Rows 1 and 4 display lower magnification images, with the images in column 1 corresponding to before laser irradiation. Two post photolysis images show an overall elevation of Ca^2+^, with an increase from blue (low Ca^2+^) to green signal (higher Ca^2+^) in IMD ratio images. We observe an increased concentration in local Ca^2+^ at regions of membrane ruffling and surrounding newly formed vesicles. Magnified insets corresponding to regions of vesicle formation are shown in rows 2, 3, 5, and 6. Ca^2+^ signal localizes to regions surrounding the vesicle during vesicle formation. Smaller vesicles are often visible at vesicle periphery (white arrows in row 6), as well as fusion of these smaller vesicles to form larger vesicles. The signal surrounding the vesicles appears to dissipate over time. The dynamic process of vesicle formation and fusion was consistently linked to elevated Ca^2+^ signal.

**FIGURE 9:**
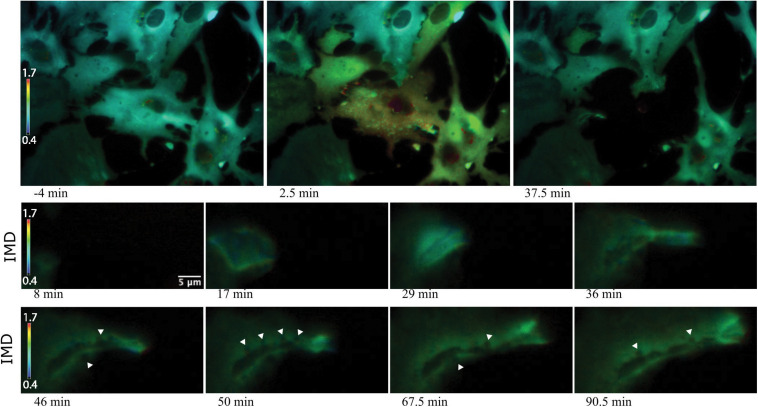
Vesicle tract formation at cell periphery coincides with increased Ca^2+^ signal. Row 1 depicts an astrocyte network in response to photolysis. Rows 2-5 magnifies an active region of a cell located at the bottom left of the lysed cell, near the cell periphery. Frequently, numerous vesicles fuse resulting in the formation of a long tract region devoid of fluorescence near the cell periphery. Smaller, vesicles localize to the edge of the tract and fuse with the central region, highlighted by the white arrows in row 4.

Imaging of Salsa6f astrocytes, requires imaging of 2 channels, cytoplasmic Ca^2+^ changes in green and reference in red for ratiometric Ca^2+^ indicator (Dong et al., 2017). Figure 7 presents a network of astrocytes at four time points: 13 min prior to photolysis, immediately after photolysis, 1 min post photolysis, and 35 min post photolysis. Figure 7 shows at each time point, a comparison of the green and red channels, G/R ratio, and intensity modulated display (IMD) ratio images for phase contrast images. GCaMP6f signal represented by the green channel shows cytoplasmic Ca^2+^ fluctuations. The Ca^2+^ insensitive tdTomato reference channel is displayed in row 2. Image processing with two methods of combined green and red ratiometric images are displayed in row 3 and 4. Green red ratio (G/R ratio) images processed and pseudo colored with look up table fire in Fiji/ImageJ software are displayed in row 3. The second processing method displayed in row 4 of Figure 7 utilizes MetaFluor software to form intensity modulated display (IMD) ratio images. The pseudo color look up table used is based on black pixels corresponding to minimum pixel intensities, increasing to blue, green, yellow, orange, red, and white at maximum pixel intensities (Tsien and Harootunian, 1990). IMD ratio results in the most dramatic Ca^2+^ signal changes. A sharp rise in Ca^2+^ fluorescence in surrounding cells is observed immediately after photolysis of the central cell, as detected by the shift in fluorescence from blue to green in the row of IMD images in Figure 7. Ca^2+^ fluorescence drops back toward baseline/pre laser exposure levels, as detected in images taken 1 min post photolysis. By 36 min post-lysis, signal within the Salsa6f photolyzed cell is absent. Lysis of the cell membrane results in the diffusion and dilution of Salsa6f into the surrounding media, resulting in the decrease in signal observed in Figure 7. Additionally, Figures 8, 9 appear to highlight the subcellular localization of Ca^2+^ with the formation of phagocytic vesicles and tracts, respectively, discussed in detail in subsequent Sections “Ca^2+^ Localization During Cell Reorientation” and “Vesicle Tract Formation.”

### Prolonged Ca^2+^ Imaging of Phagocytic Astrocytes

We examined the process of cell death in the irradiated cell with respect to changes in Salsa6f fluorescence (see Supplementary Figure S1), and morphological changes observed in matching phase contrast images. Observations during resting state (prior to laser irradiation) reveals minimal changes to mean Ca^2+^ fluorescence, other than infrequent spontaneous cytosolic waves/flashing. Photolysis results in the death of a targeted single cell, followed by a reaction from undamaged neighboring cells. The burst in cytosolic Ca^2+^ that occurs after damage to the target cell is evident by bright green and red pseudo color fluorescence throughout the cell (Refer to Supplementary Figure S1 and Supplementary Video S6). Phase contrast images show distinct cell death features such as pycnotic nuclei and phase dark cytoplasmic inclusions by 1 min following photolysis. Non-irradiated cells exhibit an increase in [Ca^2+^] as indicated by the shift from blue to green pseudo color pixel brightness. By 38 min post photolysis, surrounding cells have returned to the pre-lysis (baseline) ratio of fluorescence intensity. Subcellular resolution of Ca^2+^ signaling during the phagocytic process allows for tracking local Ca^2+^ events that are tied with morphological changes occurring during phagocytosis. This response includes a repositioning of the responding cell toward the remaining dead cell debris, and the occurrence of cytoplasmic Ca^2+^ signaling resulting the formation of endocytic vesicles, and tracts (see subsequent section).

### Ca^2+^ Localization During Cell Reorientation

**FIGURE 10:**
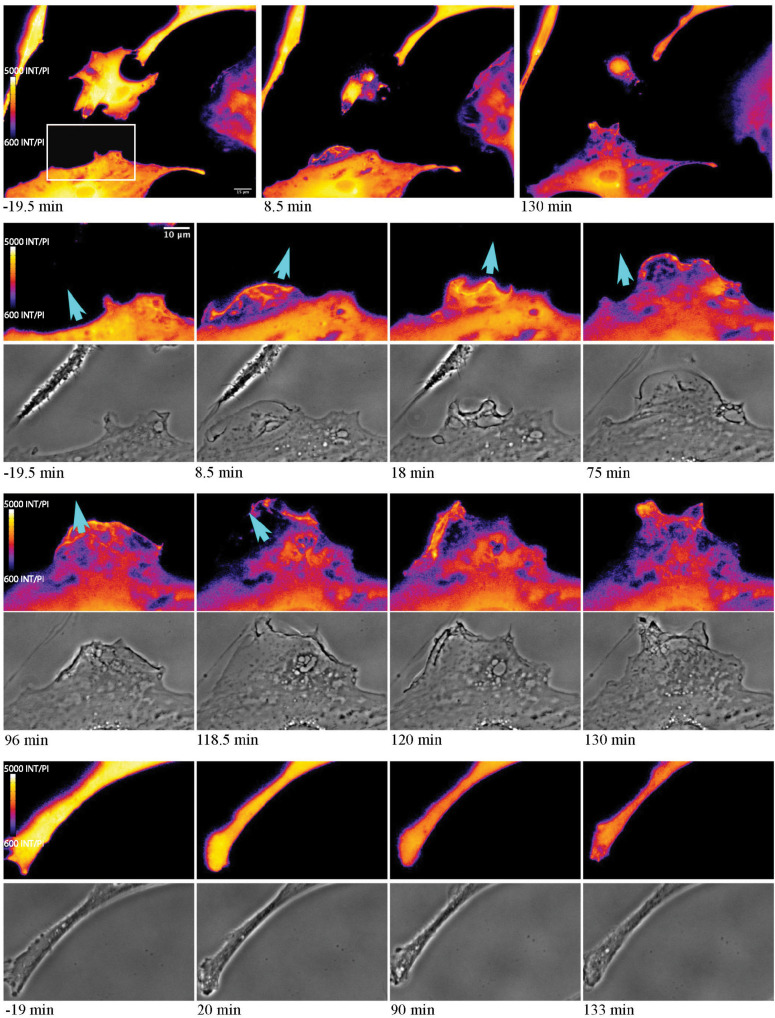
Ca^2+^ localization during cell migration toward lysed cell. Observe the reorientation and migration of the astrocyte at the bottom of the image toward the central photolyzed cell (white ROI in pre-lysis image). Rows 2–5 highlight the area depicted by the white rectangle in the pre-lysis image of row 1. As the responding cell migrates in the direction of the lysed cell (blue arrow) Ca^2+^ localizes to cell membrane ruffles and protrusions. This dynamic process continues over the 130 min imaging period following photolysis. No obvious morphology or fluorescence changes are observed in the non-responding astrocyte process, in magnified insets visible in row 6 and 7.

As astrocytes reorient and migrate toward cellular debris, a local Ca^2+^ flux occurs at the cell’s newly formed leading edge, in the direction of the remaining cellular debris (Figure 10). In response to cell death, extension of responding cells toward the dead cell debris is observed (see blue arrows in Figure 10). At 19.5 min prior to photolysis, the bottom left cell appears with a uniform orange pseudo color, with minimal Ca^2+^ changes or morphology changes prior to laser exposure. Following photolysis of an adjacent cell (ROI), increased ruffling activity occurs forming a leading edge. This can be seen in the phase contrast image and Salsa G/R ratio images. This is visible in magnified images in rows 2 through 5 of Figure 10. The color shift from dark blue to bright orange highlights the dynamic changes occurring at the newly formed leading edge as it extends its lamellae toward debris (blue arrows) at 8.5 and 130 min post photolysis (magnified insets row 2 and 4). Ca^2+^ localizes to the newly formed membrane protrusions, with increased fluorescence corresponding to the dynamic regions oriented toward the debris. Cell edges oriented away from the cellular debris (Figure 10 rows 7 and 8) do not display increases in fluorescence or morphological changes visible in phase contrast images. Here, we observe minimal changes of edge morphology and Ca^2+^ signaling, other than an overall decrease in signal over time, attributed to photobleaching of the fluorophore.

**FIGURE 11:**
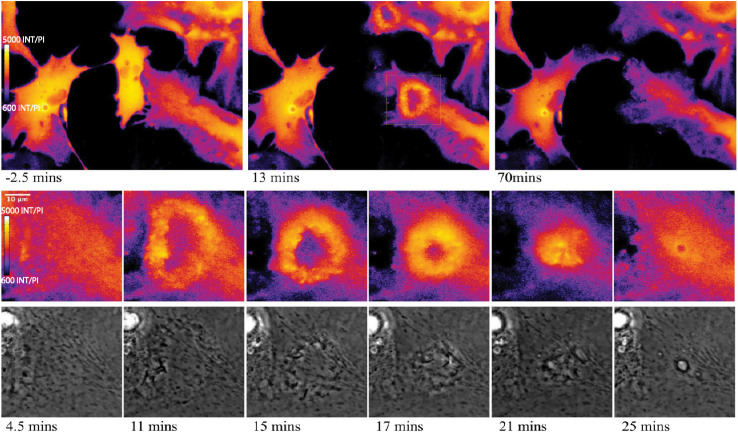
Cytoplasmic Ca^2+^ signal constriction resulting in formation of endocytic vesicle. G/R ratio images (LUT fire) highlight waves of Ca^2+^ signal in response to photolyzed cell at center. Top right and bottom right cells both display a Ca^2+^ ring visible in the 13 min post-lysis image of row 1. The rectangle in the 13 min post-lysis image of row one corresponds to the magnified insets in row 2 that track the development of vesicle formation at the center of the constricting Ca^2+^ ring. High resolution IMD images and corresponding phase contrast images highlight Ca^2+^ through the process of endocytic vesicle formation.

Of particular interest is the process of phagocytosis of the dead cell debris by the activated astrocyte (see G/R ratio pseudo color images in Figure 11 and Supplementary Video S7). Figure 11 demonstrates dramatic changes in cytosolic Ca^2+^ flux moving through responding cells. The magnified inset in row 2 highlights a concentric Ca^2+^ signal resulting in the formation of an endocytic vesicle. A uniform Ca^2+^ signal is visible throughout the first image of row 2 (magnified inset) at 4.5 min post photolysis, which dramatically changes with a formation of an oval fluorescent region at 11 min post photolysis. At 15 min post photolysis, Ca^2+^ fluorescence increases in intensity, and decreases in geometric diameter. The subcellular circular Ca^2+^ signal continues to constrict in size and increase in fluorescence as a vesicle forms. In the matching phase contrast images the vesicle is visible at 25 min post photolysis (row 3, Figure 11). Supplementary Video S7 demonstrates this concentric Ca^2+^ signal constriction occurring within 3 different locations in responding astrocytes.

We quantified the mean G/R fluorescence ratio for 3 positions: (1) the leading edge displayed in Figure 10, row 2 and 4, (2) a central cytoplasmic region 90 um from the position of laser ROI, and (3) the trailing edge not directed toward the lysed material displayed in Figure 10, row 6. For each region, G/R fluorescence ratio and the distance between the cell edge and the position of laser lysis (ROI) was graphed over time (Refer to Supplementary Figure S2). As the leading edge protrudes toward the lysed material, Ca^2+^ signal increases. By approximately 120 min, the cell slows its progression toward the lysed cell and Ca^2+^ fluorescence plateaus. This demonstrates an inverse correlation between Ca^2+^ signal and distance to ROI for the leading edge of astrocytes. The central region shows minimal changes in Ca^2+^ signal from the cell cortex. The trailing edge slowly moving away from the lysed region displayed a slight increase in Ca^2+^ signal as the astrocyte edge retracts. Ca^2+^ signal also appears to plateau around 200 min post photolysis.

**FIGURE 12:**
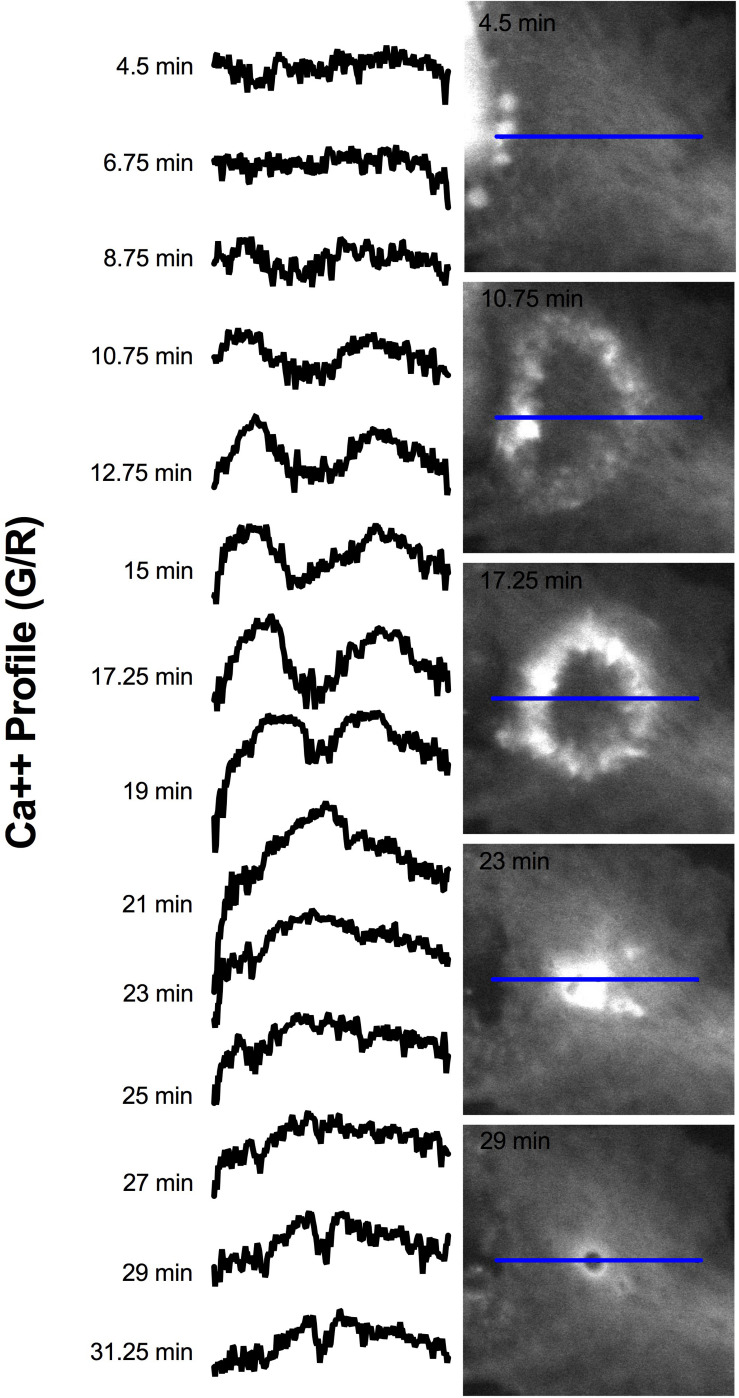
Signal profile of concentric Ca^2+^ wave constricting and resulting in phagocytic vesicle formation. G/R intensity along the blue ROI is plotted. Between 4.5 and 6.75 min following photolysis, the intensity profile remains relatively linear. Two peaks corresponding to the perimeter of the concentric Ca^2+^ circle are visible at 10.75 min, and increase in intensity through 19 min. The peaks move from the periphery of traces toward the center of the plot profile as the concentric ring constricts. The two peaks form a large central peak 21 min post photolysis, then diminishes between 23 and 27 min post photolysis. The newly formed vesicle corresponds to the small dip at the center of the plot profiles at 28 and 31.25 min.

Elevated Ca^2+^ was observed to localize in regions surrounding endocytic vesicles. Figure 12 displays the Ca^2+^ signal profile for the concentric wave as it progresses to vesicle formation. The G/R signal along the blue ROI is plotted for each time point between 4.5 and 31.25 min post photolysis. Two peaks corresponding to the perimeter of the concentric Ca^2+^ circle begin to form at 8.75 min, and increase in intensity as they move from the periphery toward the center of the blue ROI. The two peaks meet to form a large central peak at 21 min post photolysis, then diminishes as an endocytic vesicle forms. The position of the vesicle is visualized as a small dip at the center of the trace at 28 and 31.25 min. A small amount of fluorescence surrounding the endocytic vesicle remains following vesicle formation. Figure 8 depicts the localization of Ca^2+^ during vesicle formation and maturation in two different astrocyte populations.

### Vesicle Tract Formation

Some responding astrocyte with high phagocytic activity formed tracts of vesicles devoid of fluorescence at the cell periphery (Figure 9). Multiple small vesicles (white arrowheads) devoid of fluorescence combine and form a high dynamic region where a central track is visible, devoid of Ca^2+^. Elevated Ca^2+^ levels around the tract persist. IMD ratio images resolve small vesicles that continue to migrate to the periphery of the tract and fuse with the central cavity (see white arrows in Figure 9, rows 5 and 6).

## Discussion

In the present study, we examine changes in cytosolic [Ca^2+^] within astrocytes: (1) as an early response to nearby cell damage/death; and (2) during subsequent phagocytosis. We have shown that astrocytes from the adult Salsa6f mouse, embryonic E18 mice (Brain Bits), and from an established Ast1 cell line, all respond with a significant increase in intracellular cytosolic Ca^2+^ in response to a killed or severely damaged nearby astrocyte. This is consistent with perturbations of astrocytes observed in previous publications, including Kanemaru et al. (2013) that showed both an immediate and persistent (3 h) elevation of Ca^2+^ in the neocortex region mostly composed of astrocytes following laser induced injury *in vivo*. It is common to observe spontaneous oscillation in Ca^2+^ signal in astrocytes; these have been published in numerous studies. There are many known external stimuli resulting in elevated cytoplasmic Ca^2+^ concentrations and both synchronized and random oscillations including gliotransmitters (Jourdain et al., 2007), neuronal activity (Takata et al., 2011; Min and Nevian, 2012), mechanical stress (Verkhratsky and Kettenmann, 1996), and ATP (Guthrie et al., 1999; Cotrina et al., 2000; Neary et al., 2003). With so many potential external stimuli that are known to evoke an elevated Ca^2+^ response, residual peaks could be due to many potential cellular signaling changes that occur as astrocytes respond to the induced damage. Our findings support a functional role of Ca^2+^ as an important signaling component that travels through the astrocyte network in response to injury. It is likely the increase in Ca^2+^ signaling is due to the release of small purinergic compounds, like ATP, diffusing from the photolyzed cell and interacting with the observed astrocytes. ATP or other small signaling molecules could serve as an intermediate signaling factor which triggers both the immediate Ca^2+^ transients and phagocytosis observed in the astrocytes observed in this study. Understanding the specific signaling mechanisms of the observed astrocyte response is of great importance and will be the focus of future studies.

### Ca^2+^ Signal Transmission Through Astrocyte Network

In addition to the occurrence of the Ca^2+^ oscillation in the cell attached to the photolyzed cell, we also observed a rapid transmission of the Ca^2+^ oscillation through networks of attached astrocytes. This cellular model allowed us to monitor spatial and temporal Ca^2+^ dynamics throughout a network of astrocytes as they respond to cell injury and death. Attached, networked, and isolated astrocytes exhibited transients in intracellular [Ca^2+^] following laser induced death of an adjacent cell. Ca^2+^ levels returned to levels similar to pre-lysis. The increase in intracellular Ca^2+^ observed throughout the contiguous astrocyte network (attached and networked cells) are likely due to signaling through intercellular gap junctions that directly link the cytoplasm of neighboring cells. The functional role of gap junction in Ca^2+^ movement between astrocytes has been well documented in previous studies (Enkvist and McCarthy, 1992; Finkbeiner, 1992; Venance et al., 1998). The variation in Ca^2+^ dynamics observed in isolated astrocytes *in vitro* differed greatly from attached and networked astrocytes that were contiguous with the damaged cell. Small molecules cannot move through normally present intercellular junctions, thus removing one physical mechanism to induce a Ca^2+^ response in the observed astrocyte.

This result was unlike the elevation in Ca^2+^ observed by Morizawa et al. (2017) lasting days compared to seconds/minutes that was observed here. Our *in vitro* model allowed for the quantification of recovery time based on the proximity of the responding cell to the injury. Observed time to recovery toward the pre-lysis [Ca^2+^] levels varied depending on the proximity of the responding astrocyte to the damaged cell (attached, networked, or isolated) (Figure 3, *T*_1__/__2_ ranged 25–60 s).

The temporal kinetics of the Ca^2+^ dynamics are similar for astrocytes from all three sources with the exception that isolated Ast1 cells exhibited a strong Ca^2+^ oscillation after the killing event, whereas isolated cells from primary cultures had only small Ca^2+^ transients. The fact that isolated cells respond to the killing event suggests that diffusible elements such as ATP from the killed cell initiates a Ca^2+^ signaling cascade within isolated cells, possibly via purinergic receptors on the cell surface (Fujii et al., 2017). Isolated cells from the established cell line appear to be very susceptible to these changes. This observation, and the potential role purinergic compounds and receptors play in detecting cellular damage by astrocytes will be followed up in future studies. This cellular model is perfect for the application of inhibitors and the observation of any ensuing changes to Ca^2+^ in response to cellular injury.

Some variability in individual cell response was observed, and may be related to the state of activation of individual cells. Previous studies have shown heterogeneity in astrocyte function, reactivity, and even prevalence of the common astrocyte marker GFAP (Sofroniew and Vinters, 2010). Notwithstanding theses variables, most of the individual astrocytes responded with a significant Ca^2+^ oscillation following the photolysis event, which subsequently recovered toward the pre-lysis baseline value. Fluorescent images of multicellular networks show that some cells in the network undergo increases in the frequency of Ca^2+^ transients. The small percentage of cells responding in this manner provides further evidence of variability in individual cell response within an astrocyte network.

### Ca^2+^ Signaling During Phagocytosis

The use of genetically encoded Ca^2+^ indicators provide increased potential for visualizing Ca^2+^ dynamics during cellular processes. Salsa6f was incorporated into the genome of a mouse and targeted astrocytes via GFAP, which permits observation of Ca^2+^ changes only in astrocytes throughout the responding astrocyte networks. Salsa6f labeling provides a significant advantage over chemical indicator Fluo4 that indiscriminately labels all cell types within a culture. An additional advantage of Salsa6f astrocytes is that cultures remained fluorescent and viable for an extended period of time. This permitted monitoring of Ca^2+^ dynamics for extensively longer imaging periods without the addition of exogenous chemical indicators. Fluo4 was better at monitoring large scale changes in cytosol with increased dF/F in tracking adjacent cell’s response to cell death, however photobleaching and deleterious effects of Fluo4 on cells was not a factor with Salsa6f labeled cells. Salsa6f cultures allowed for acquisition of hundreds of images with minimal photobleaching.

High resolution optical imaging of *in vitro* astrocytes revealed elevated Ca^2+^ levels throughout the phagocytic process. Salsa6f, a recently developed Ca^2+^ fluorescent probe Salsa6f (Dong et al., 2017), was an ideal indicator for imaging local events of astrocytes, revealing subcellular localization during multiple steps of the phagocytic process, including lamellae formation, endocytic vesicle formation, vesicle maturation, and vesicle tract formation.

This study establishes the involvement of Ca^2+^ in the process of endocytosis of cellular debris described in our previous paper (Wakida et al., 2018). This observation is consistent with a role for astrocytes in removing cellular debris from the CNS following injury such as in traumatic brain injury (Sofroniew, 2009). Similar to our observations, Nunes and Demaurex (2010) reported Ca^2+^ elevation at phagocytic sites in mouse embryonic fibroblasts. Astrocyte phagocytosis likely occurred through a similar process. One possible role of Ca^2+^ localization is to trigger the rearrangement of the cytoskeleton, as cytoskeletal rearrangement is necessary for all major phagocytic steps observed with highly localized Ca^2+^ transients. Actin polymerization is necessary for the major steps of phagocytosis, including membrane protrusion to the targeted debris, particle engulfment, and formation of the phagocytic cup (Rougerie et al., 2013). Future studies should focus on the molecular mechanisms responsible for local Ca^2+^ events during the phagocytosis.

A potential mechanism for the localized Ca^2+^ dynamics may involve the same receptor pathways observed during synapse elimination such as those initiated via Mer proto-oncogene tyrosine kinase (MERTK) and Multiple EGF-Like Domains (MEGF10). Phosphatidylserine recognition has been shown to be a key step during the initiation of phagocytosis (Chang et al., 2000; Jung and Chung, 2018). During photolysis, cells expose phosphatidylserine to surrounding astrocytes. Phagocytic receptors MERTK and MEGF10 are expressed in astrocytes and may bridge with phosphatidylserine from the dead cell (Stitt et al., 1995; Cahoy et al., 2008; Zhang et al., 2014). MERTK can activate phospholipase C γ2 which can lead to DAG and IP3 production, thus triggering Ca^2+^ release and PKC mediated Rac1 cytoskeletal rearrangement (Todt et al., 2004). Additionally, a recent study demonstrated Rac1 potentiated ORAI1 translocation to the leading edge of migrating cells (Lopez-Guerrero et al., 2020). The localized Ca^2+^ dynamics observed in our study are likely due to cytoskeletal remodeling during astrocyte phagocytosis. The specific signaling mechanism linking the Ca^2+^ transients and cytoskeletal remodeling will be the focus of follow-up studies.

### Origin of Ca^2+^ Transients

It was important to elucidate the source of the immediate transient increase in cytosolic Ca^2+^ signal, especially considering its role as a possible secondary messenger. Oscillations from cells in Ca^2+^ free and low Ca^2+^ medium show that the transient increase in cytosolic Ca^2+^ is likely due to release from internal stores. Our results showing Ca^2+^ transients in isolated cells support this. A common feature of metabotropic receptor induced Ca^2+^ transients is the ability to persist in Ca^2+^ free extracellular solutions (Verkhratsky et al., 2012). Therefore, the initial transient is likely resulting from a purinergic stimulated IP3 induced Ca^2+^ release. Interestingly, Ast1 in low Ca^2+^ medium exhibited transients with larger dF/F than cells in regular media or Ca^2+^ free medium. Previous studies have shown that despite efforts to completely eliminate Ca^2+^ contamination during the making of Ca^2+^ free solutions, a remainder of approximately 15 μM was still found in solution as determined by absorption spectroscopy (Lucas et al., 1990). Our low Ca^2+^ medium did not contain EGTA and as a result may have included free Ca^2+^ ions available which could undergo influx. It is likely that externally low Ca^2+^ levels do not provide enough feedback to stop influx or Ca^2+^ release from internal stores. Cells in EGTA had lower dF/F when compared to cells in regular and low Ca^2+^. These results suggest that ER Ca^2+^ may have been slowly depleting and thus the transient had a smaller dF/F. Extracellular Ca^2+^ fluctuations can lead to changes in Ca^2+^ concentration within stores as cells attempt to modulate external Ca^2+^ concentrations (Hofer, 2005). Furthermore, the ability of cells in Ca^2+^ free medium to respond to a second cell lysis demonstrates that some of the Ca^2+^ which was released may have been re-sequestered and released or that the stores were not fully depleted during the first oscillation.

The duration (*T*_1__/__2_) of the oscillation was lower when Ca^2+^ dF/F peak amplitude was larger. Since the activity of pumps and channels is modulated by the level of cytoplasmic Ca^2+^, it seems that different mechanisms are leading to the drop in cytoplasmic Ca^2+^ within our experiments (Bagur and Hajnoczky, 2017). Therefore, cells in low Ca^2+^ medium whose transients are larger than in regular medium are likely activating the sodium Ca^2+^ exchanger (NCX) and mitochondrial Ca^2+^ uniporter (MCU). Whereas the cells in Ca^2+^ containing medium may primarily rely on the higher affinity plasma membrane Ca^2+^ transport ATPase (PMCA) and sarco/endoplasmic reticulum Ca^2+^ ATPase (SERCA) pumps to return the cell to low cytoplasmic Ca^2+^ levels.

In summary, we demonstrate changes in intracellular Ca^2+^ ions are involved in the phagocytic response of astrocytes. Selective killing of a single astrocyte results in a rapid increase in intracellular Ca^2+^ in astrocytes adjacent to the photolyzed cell. This increase is most likely due to release of Ca^2+^ from internal stores. Studies were performed on astrocytes from three different sources, using two different Ca^2+^ sensitive fluorescent probes to monitor changes in intracellular Ca^2+^. Ca^2+^ changes correlated with changes in cell morphology, including leading edge protrusions, migration, and concomitant formation of endocytic vesicles characteristic of phagocytosis of the dead cell corpses.

## Data Availability Statement

The datasets presented in this study can be found in online repositories. The names of the repository/repositories and accession number(s) can be found below: figshare 10.6084/m9.figshare.12021078.

## Ethics Statement

The animal study was reviewed and approved by the University of California, Irvine, Institutional Animal Care and Use Committee. Written informed consent was obtained from the owners for the participation of their animals in this study.

## Author Contributions

NW, VG-G, MB, and MC designed the experiments for this study. NW, VG-G, HL, JN, EK, PD, LS, and CC executed the experiments, data analysis, and statistical testing. SO developed the Salsa6f mouse. AL provided the mouse tissue preparations. LS developed the software for laser microscopy system. LT, JD, MC, and MB provided the critical feedback and direction of project. MB, NW, VG-G, HL, and PD wrote the manuscript. All the authors contributed to the article and approved the submitted version.

## Conflict of Interest

The authors declare that the research was conducted in the absence of any commercial or financial relationships that could be construed as a potential conflict of interest.
