# Supplementary material for: Calcium Dynamics in Astrocytes During Cell Injury
Source: Front Bioeng Biotechnol. 2020 Aug 27;8:912. doi: 10.3389/fbioe.2020.00912 (PMC7481337; doi:10.3389/fbioe.2020.00912)
Supplement: Supplementary file 8 [file Data_Sheet_1.PDF]

Supplementary Material

Supplementary Video 1. Ca^{2+} elevation in Fluo4 labeled established astrocyte cells following photolysis (G/R ratio images). Astrocytes spontaneously fire during a resting state prior to photolysis (time -75 s to time 0 s). Immediately following photolysis, astrocytes throughout the network spike in cytosolic Ca^{2+}. Fluorescence levels decrease to baseline levels observed prior to photolysis.

Supplementary Video 2. Ca^{2+} elevation in Fluo4 labeled primary astrocytes following photolysis (G/R ratio images). Primary astrocytes surrounding the central targeted cell spike in Ca^{2+} fluorescence in response to photolysis.

Supplementary Video 3. Ca^{2+} elevation in Salsa6F labeled primary astrocytes following photolysis (G/R ratio images). Synchronized Ca^{2+} cytosolic spikes occur in non-induced cells prior to photolysis. Elevation in Ca^{2+} throughout the astrocyte network in response to photolysis of a central cell is observed.

**dF/F p values**	**Fluo4/Ast1**	**Fluo4/Primary**	**Salsa6F**
Control vs. Attached	<0.01	0.03	<0.01
Control vs. Networked	<0.01	0.04	<0.01
Control vs. Isolated	0.01	0.02	0.02
Attached vs. Networked	0.71	0.16	0.01
Attached vs. Isolated	0.34	<0.01	<0.01
Networked vs. Isolated	0.21	0.02	<0.01

Supplemental Table 1. Statistical comparison of dF/F values between astrocyte network categories.

Supplemental Video 4. Salsa6F astrocytes responding to the photolysis of an attached cell with an increase in frequency of Ca^{2+} spikes (IMD ratio images).

Supplemental Video 5. Time lapse movie of perfused primary Salsa6F astrocytes responding to two photolysis events in Ca^{2+} free medium (IMD ratio images).

Supplemental Figure 1. Progression of death of the photolysed cell and reaction of surrounding Salsa6F astrocytes. A bright green and red burst in cytosolic Ca^{2+} occurs in the targeted cell as a result of photolysis (IMD ratio images). Morphological changes are depicted in corresponding phase contrast images. Phase contrast display cell death features in irradiated cells, including pyknotic nuclei and dark cytoplasmic inclusion.

Supplemental Video 6. Time lapse video of attached Salsa6F cells (IMD ratio images) responding to photolysis by the formation of endocytic vesicles.

Supplemental Video 7. Time lapse (G/R ratio images) demonstrating concentric Ca^{2+} signal constriction in 3 different locations in responding Salsa6F astrocytes.
