# Supplementary figures and images for: Calcium Dynamics in Astrocytes During Cell Injury
Source: Front Bioeng Biotechnol. 2020 Aug 27;8:912. doi: 10.3389/fbioe.2020.00912 (PMC7481337; doi:10.3389/fbioe.2020.00912)

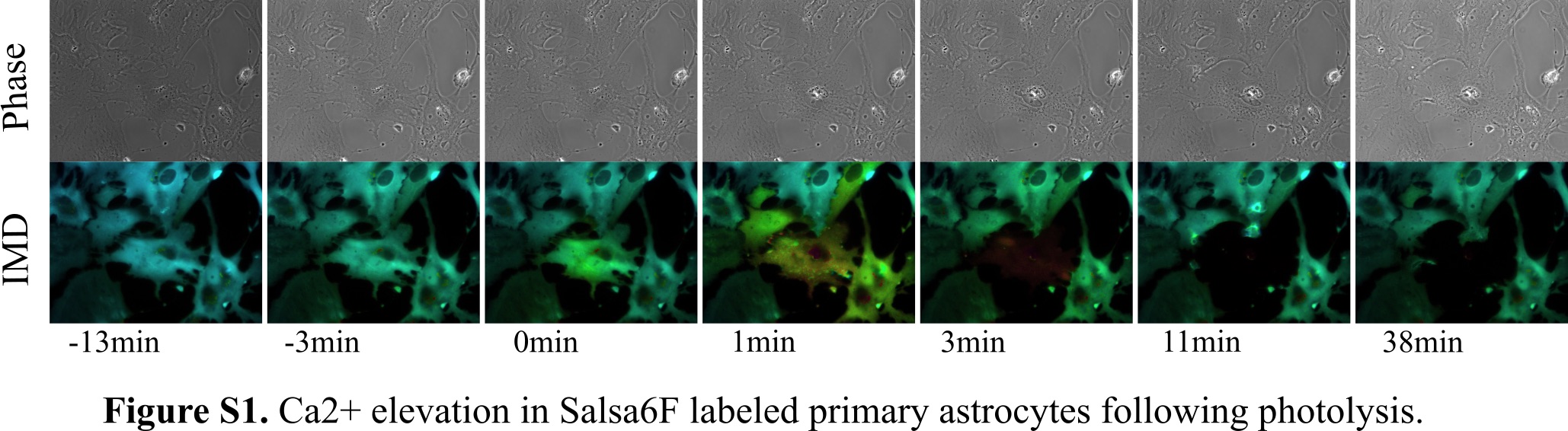

Supplement: Supplementary file 9 [file Image_1.JPEG]

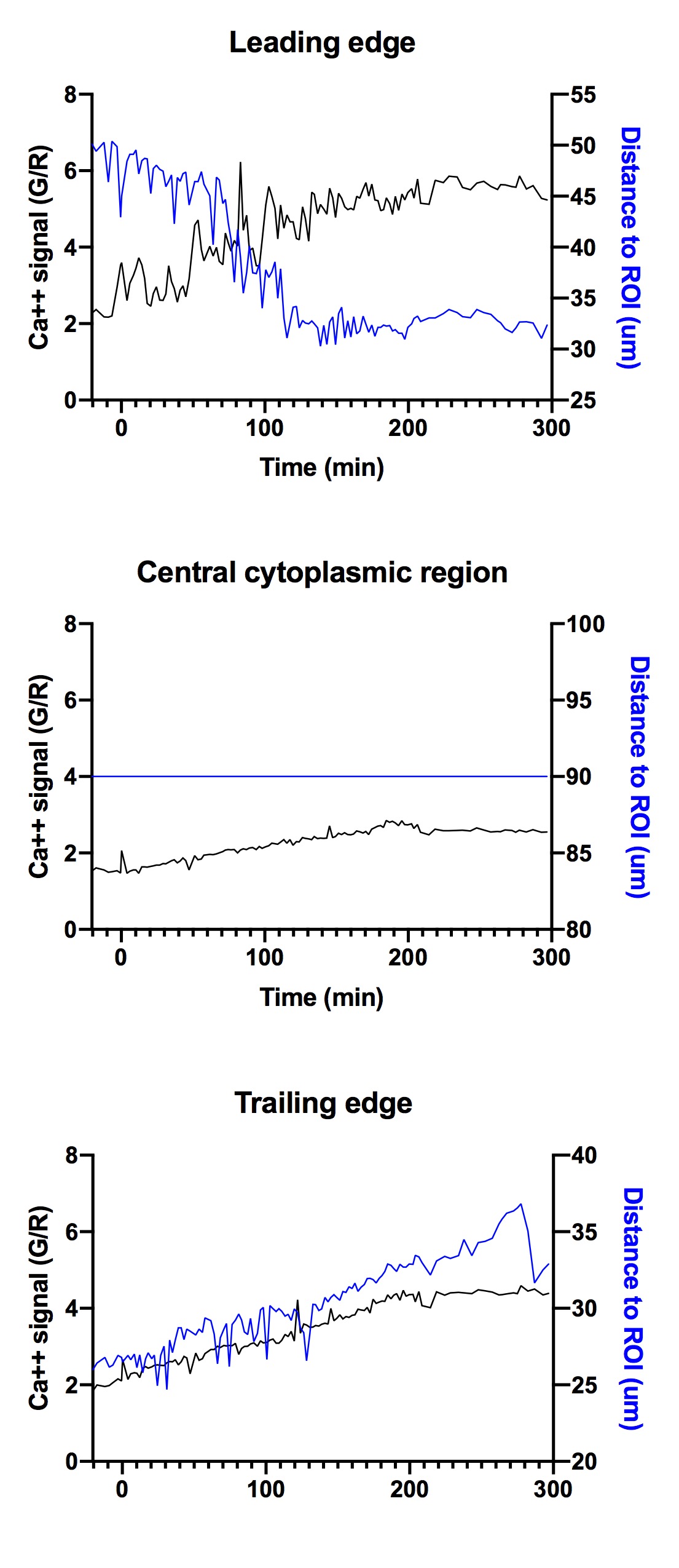

Supplement: Supplementary file 10 [file Image_2.JPEG]
